# Identification of DNA methylation prognostic signature of acute myelocytic leukemia

**DOI:** 10.1371/journal.pone.0199689

**Published:** 2018-06-22

**Authors:** Haiguo Zhang, Guanli Song, Guanbo Song, Ruolei Li, Min Gao, Ling Ye, Chengfang Zhang

**Affiliations:** 1 Department of Hematology, Qilu Hospital, Shandong University, Jinan, Shandong, P.R. China; 2 Department of Hematology, Jining NO.1 People’s Hospital, Jining, Shandong, P.R. China; 3 Department of Preventive and Health Care, Guang’anmen Hospital, China Academy of Chinese Medical Sciences, Beijing, P.R. China; 4 Department of Clinical Laboratory, Jining Chinese Medicine Hospital, Jining, Shandong, P.R. China; 5 Department of Clinical Laboratory, Jining NO.1 People’s Hospital, Jining, Shandong, P.R. China; INSERM1163, FRANCE

## Abstract

**Background:**

The aim of this study is to find the potential survival related DNA methylation signature capable of predicting survival time for acute myelocytic leukemia (AML) patients.

**Methods:**

DNA methylation data were downloaded. DNA methylation signature was identified in the training group, and subsequently validated in an independent validation group. The overall survival of DNA methylation signature was performed. Functional analysis was used to explore the function of corresponding genes of DNA methylation signature. Differentially methylated sites and CpG islands were also identified in poor-risk group.

**Results:**

A DNA methylation signature involving 8 DNA methylation sites and 6 genes were identified. Functional analysis showed that protein binding and cytoplasm were the only two enriched Gene Ontology terms. A total of 70 differentially methylated sites and 6 differentially methylated CpG islands were identified in poor-risk group.

**Conclusions:**

The identified survival related DNA methylation signature adds to the prognostic value of AML.

## Introduction

Acute myeloid leukemia (AML) is a highly aggressive hematologic malignancy characterized by a vast proliferation of immature myeloid blasts that accumulate in the bone marrow and blood. AML is closely correlated to cytokine networks of proliferation, differentiation and apoptosis of leukemic cells [1]. AML is caused by different factors including radiation, mutations and carcinogens [2–5]. The disease can progress quickly and can become fatal in a short period of time without treatment. Known prognostic factors of AML include age, mutations, complex karyotype, the antecedent hematologic disease, presence of elevated white blood cell counts and prior chemo or radiotherapy for another malignancy [6]. Intensive chemotherapy is initially effective in most patients with AML, however, the surviving LIC clones repopulate the disease and lead to subsequent disease relapse and poor prognosis [7]. In addition, the treatment of elderly patients and patients with relapsed refractory remains a challenge [8–10]. Therefore, an improved understanding of the molecular mechanisms underlying of AML could be helpful to improve the treatment efficacy to prolong the survival time for patients.

DNA methylation is an important epigenetic mechanism in regulating gene expression [11]. It is pointed out that epigenetic disturbances have been involved in the pathogenesis of leukemia [12]. Jiang Y et al found that progression from myelodysplastic syndrome to AML was correlated to increased aberrant DNA methylation [13]. Previous studies have investigated genome-wide methylation in AML [14]. In AML, the presence of common methylation patterns in *p15* and E-cadherin has been described [15, 16]. In addition, methylation of secreted frizzled related protein (*sFRP)1*, *sFRP12*, *sFRP13* and *sFRP15* with corresponding transcriptional silencing has been found in AML cell lines [17]. The survival analysis showed that GATA binding protein 4 (*GATA4*) promoter methylation was significantly associated with shorter overall survival of pediatric AML [18]. Thus it can be seen that DNA methylation may play a crucial role in the development of AML. In this study, we aimed to find potential survival-related DNA methylation signature in AML, which may pave the way for the development of novel tumor markers and therapeutic targets for AML.

## Materials and methods

### DNA methylation data retrieval and analysis

DNA methylation data were downloaded from the TCGA dataset (http://tcga-data.nci.nih.gov/tcga). The data was derived from blood of AML. Among which, there were 195 samples with DNA methylation information. There were 188 samples with follow-up information. Finally, we selected 182 samples with both DNA methylation information and follow-up information. In order to improve data accuracy, the DNA methalytion sites were first preprocessed. DNA methalytion sites on sex chromosomes were excluded. Considering the heterogeneity of blood, based on the DNA methylation data, blood components were then predicted using the R packages in RefFreeEWAS. Finally, all samples were divided into the training group (127 cases) and validation group (55 cases) randomly. There were no overlapped cases between two groups. The chi-square test and t-test were used to analyze the statistical difference of clinical index between the two groups. And there was no significant difference in age, race, gender, vital status, survival time and disease risk result between the two groups. The clinical characteristics of these two groups were shown in Table 1.

**Table 1:** The clinical characteristics for training group and validation group.

Clinical index		Training group (n = 127)	Validation group (n = 55)	P value
**Age**	**Mean ± SD**	55.28346±16.05244	55.65455±16.52632	0.8886992
	**Median**	59	58	
**Race**	**Asia, Black or African**	1	1	0.4152429
	**American**	6	5	
	**White**	119	48	
	**NA**	1	1	
**Gender**	**Female**	54	29	0.268031
	**Male**	73	26	
**Vital status**	**Alive**	50	14	0.1017496
	**Dead**	77	41	
**Survival time**	**Mean ± SD**	543.4724± 601.9671	570.3091 ±539.5071	0.7667396
	**Median**	334	365	
**Disease risk**	**Favorable**	26	9	0.7268791
	**Intermediate/Normal**	73	33	
	**Poor**	25	13	

NA: Not applicable.

### Identification of survival related DNA methylation sites

In order to select the survival associated DNA methylation sites, all DNA methylation sites were analyzed by the single factor Cox proportional hazard (CoxPH) regression after adjustment of age, race, gender, blood constituent and cytogenetic risk. Similarly, after further adjustment of age, race, gender, blood constituent and cytogenetic risk, the multi-factor CoxPH regression analysis was used for identification of DNA methylation signature in the survival evaluation. The statistical significance was set at p<0.001. In order to investigate the characteristic of DNA methylation signature, the Illumina Infinium HumanMethylation450 BeadChips Assay was utilized for DNA methylation sites annotation.

### Functional analysis of survival related DNA methylation signature genes

In order to study the biological function of survival-related DNA methylation signature genes, the Gene Ontology (GO) and Kyoto Encyclopedia of Genes and Genomes (KEGG) pathway analysis were performed by using the online software GeneCodis3 (http://genecodis.cnb.csic.es/analysis). And the threshold of false discovery rate (FDR) < 0.05 was set as the criteria of statistical significance.

### Survival time analysis of DNA methylation signature in the training group and validation group

The risk score (RS) of identified DNA methylation signature was calculated by the following equation:

$$Risk\;Score\;(RS)=\sum_{i=1}^{N}\;(M{\text{eth}}_{i}*C_{i})$$

N presents the number of DNA methylation; Methi presents the spectrum of ith DNA methylation site; Ci represents regression coefficient of ith DNA methylation site in the multi-factor CoxPH regression analysis. Kaplan-Meier survival curves were drawn and compared among subgroups using log-rank tests.

In addition, the receiver operating characteristic (ROC) analysis was performed to assess the 5 years’ survival value of DNA methylation signature by using pROC package in R language. The area under the curve (AUC) under binomial exact confidence interval was calculated and the ROC curve was generated.

### Identification of risk related DNA methylation sites

In order to identify the risk-related DNA methylation sites between patients with poor-risk (38 cases) and favorable-risk (35 cases), the related DNA methylation data were downloaded. We selected CpG sites based on differential methylation value calculated as mean (β case) − mean (β normal) (Δβ) combined with the false discovery rate (FDR) values. Finally, the threshold of |Δβ|>0.2 and FDR<0.05 was set as the criteria of statistical significance.

## Results

### Survival related DNA methylation signature

After original data preprocess, a total of 3884 DNA methylation sites were identified in the training group. These DNA methylation sites were used for the single factor and multi-factor CoxPH regression analysis. The result showed that 8 DNA methylation sites were identified. 8 DNA methylation sites were located in 4 CpG islands (chr12:81102034–81102716, chr17:78863569–78863813, chr3:10183305–10183941 and chr6:29600192–29600661) and 6 genes (*MYF6*, *RPTOR*, *MMP10*, *SH3PXD2B*, *VHL* and *GABBR1*). The annotation of 8 DNA methylation sites was shown in Table 2.

**Table 2:** The annotation of 8 DNA methylation sites.

Site	Strand	Gene	Gene context	CpG island	CpG island context
cg26400830	F				
cg20171297	R	*MYF6*	1stExon; 5'UTR	chr12:81102034–81102716	N_Shore
cg09891288	R	*RPTOR*	Body; Body	chr17:78863569–78863813	Island
cg02061229	R	*MMP10*	Body		
cg19979108	R	*SH3PXD2B*	Body		
cg20916523	R	*VHL*	Body; Body	chr3:10183305–10183941	S_Shore
cg21644740	F	*GABBR1*	Body; Body	chr6:29600192–29600661	N_Shore
cg26182859	R				

### Functional annotation of survival related DNA methylation signature genes

In order to further study the biological function of survival-related DNA methylation signature genes (*MYF6*, *RPTOR*, *MMP10*, *SH3PXD2B*, *VHL* and *GABBR1*), GO and KEGG functional annotation were performed. The result showed that only 2 GO terms were obtained. Protein binding was the most significantly enriched molecular function (FDR = 0.0103025) involving *RPTOR*, *VHL* and *SH3PXD2B*; cytoplasm (FDR = 0.00664319) was the most significantly enriched cellular component involving *RPTOR*, *GABBR1*, *VHL* and *SH3PXD2B*. Enriched GO terms of survival related DNA methylation signature genes were shown in Table 3.

**Table 3:** Enriched GO terms of survival related DNA methylation signature genes.

Items	Items_Details	P value	FDR	Genes
GO:0005515	protein binding (MF)	0.00515125	0.0103025	*RPTOR*,*VHL*,*SH3PXD2B*
GO:0005737	cytoplasm (CC)	0.00664319	0.00664319	*RPTOR*,*GABBR1*,*VHL*,*SH3PXD2B*

FDR: false discovery rate

MF: molecular function

CC: cellular component

### Survival time analysis of DNA methylation signature

**Fig 1:**
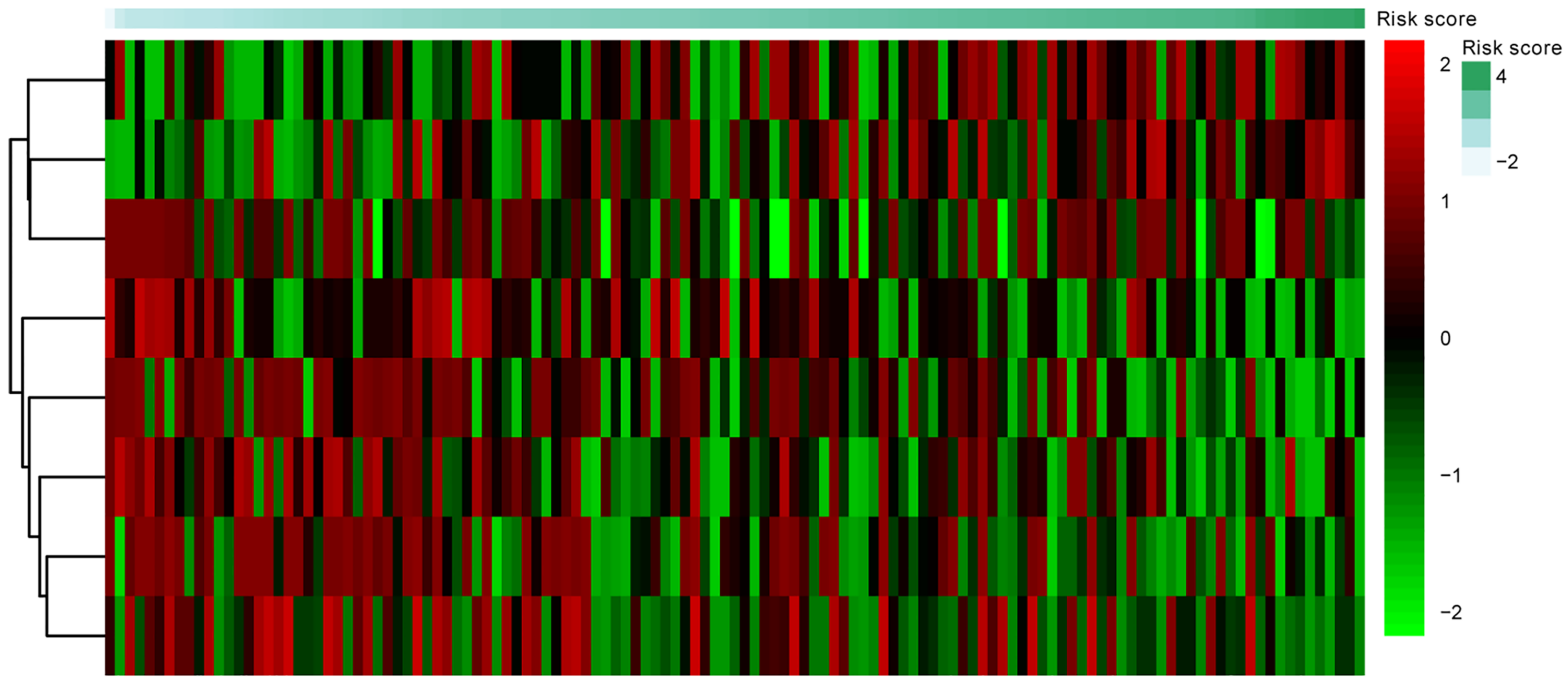
The clustering analysis map of methylation value in the DNA methylation signature sites. Diagram presents the result of a two-way hierarchical clustering of DNA methylation sites and risk score. The clustering is constructed using the complete-linkage method together with the Euclidean distance. Each row represents a DNA methylation site, and each column, a risk score value. The risk score clustering tree is shown on the right. The colour scale illustrates the relative value of the risk score: red, below the reference channel; green, higher than the reference.

**Fig 2:**
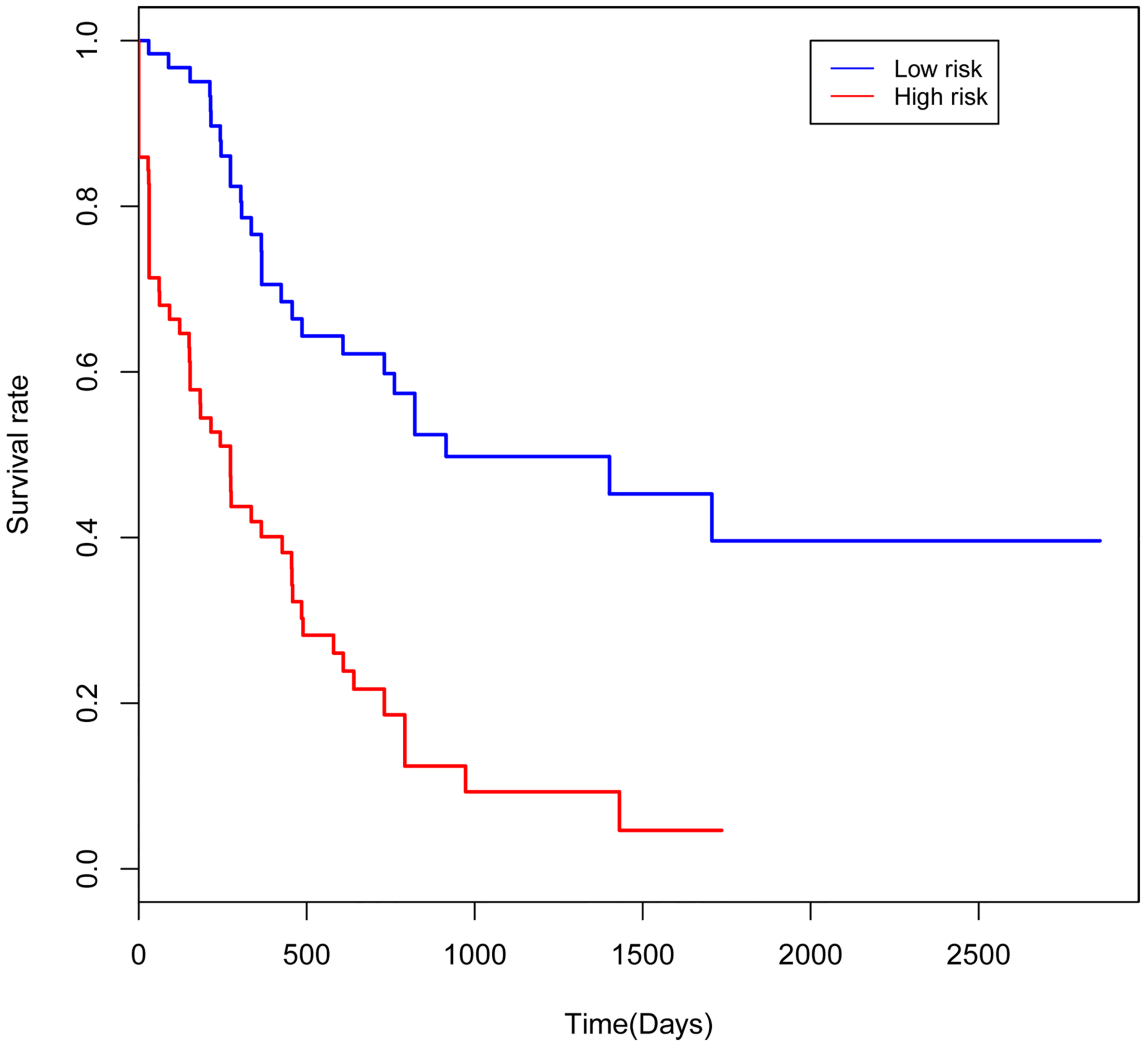
Kaplan-Meier curves showing AML patients dichotomized based on risk score in the training group. High risk is defined as a risk score ≥ the median, and low risk is defined as a risk score < the median in the training group.

**Fig 3:**
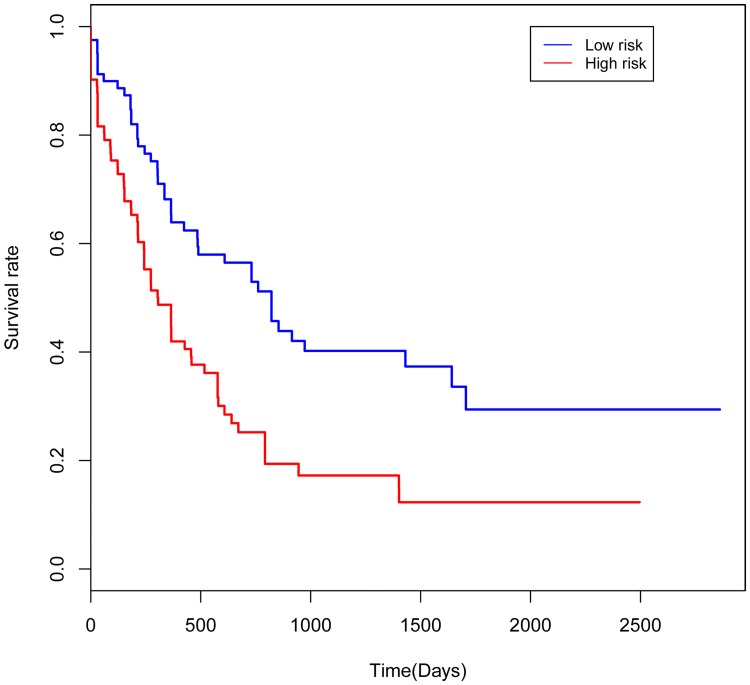
Kaplan-Meier curves showing AML patients dichotomized based on risk score in the validation group. High risk is defined as a risk score ≥ the median, and low risk is defined as a risk score < the median in the validation group.

**Fig 4:**
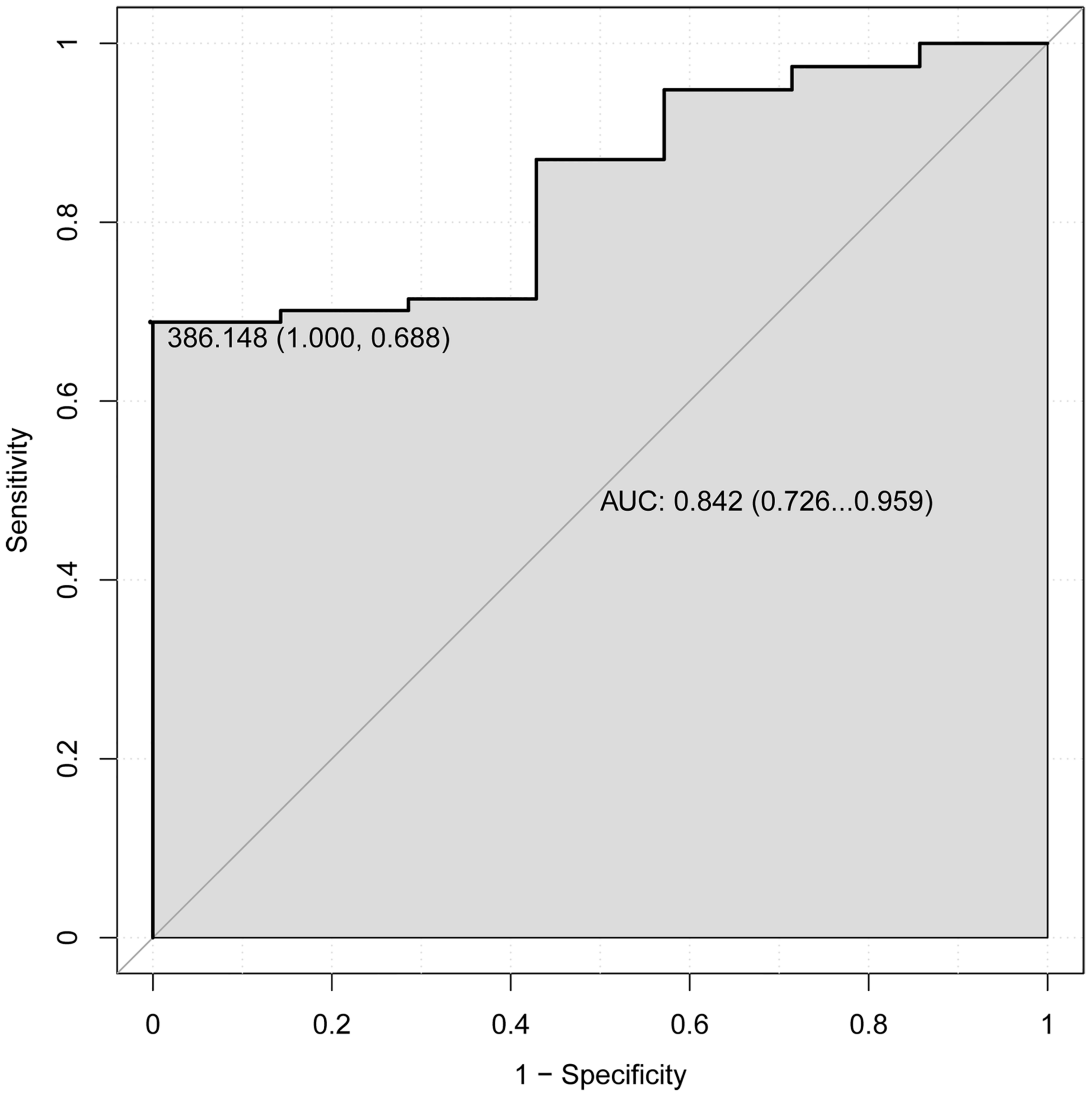
5 years’ ROC curves of DNA methylation signature in the training group. The ROC curves were used to show the diagnostic ability of DNA methylation signature with 1-Specificity and sensitivity. The x-axis shows 1-specificity and y-axis shows sensitivity.

**Fig 5:**
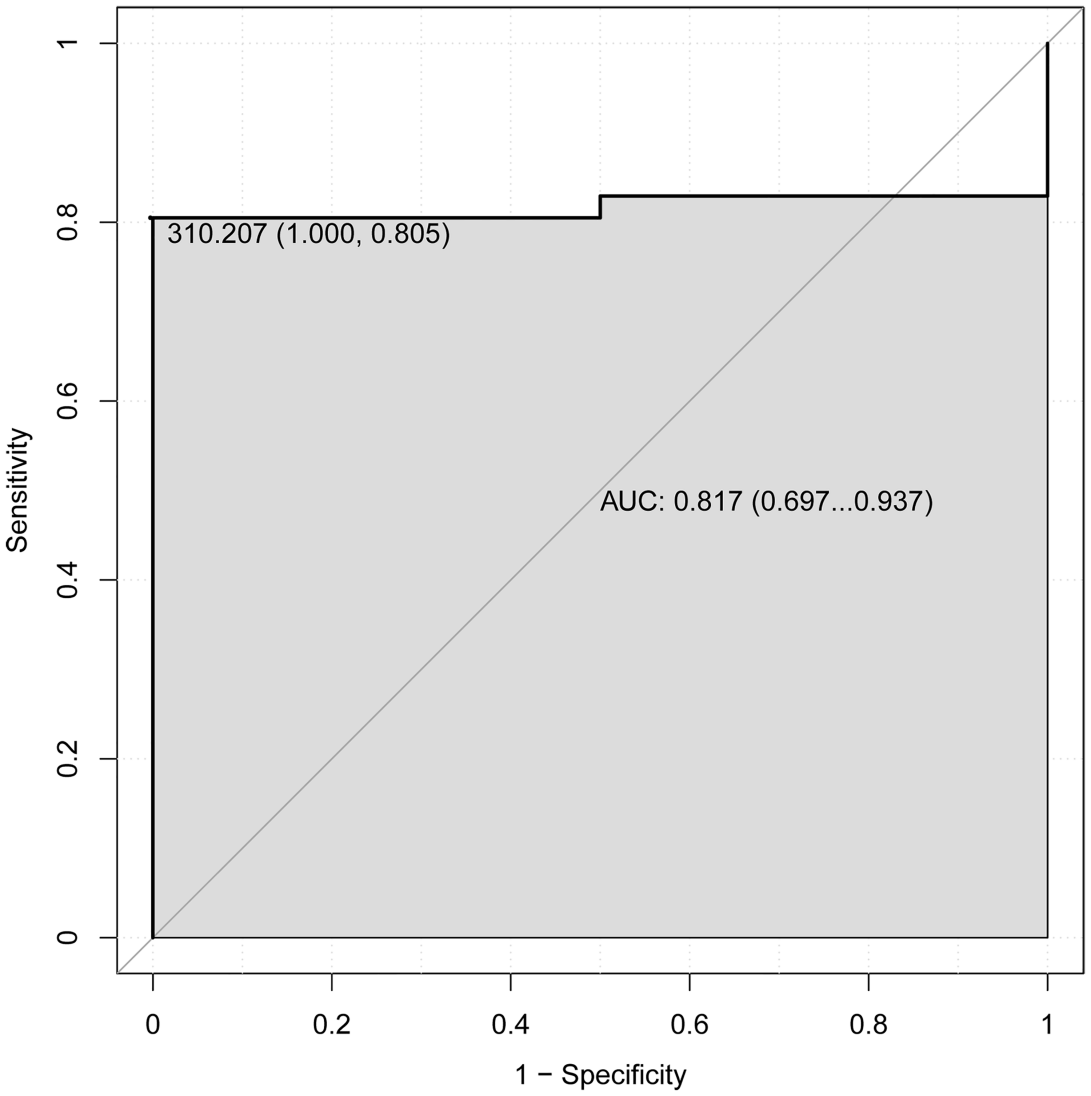
5 years’ ROC curves of DNA methylation signature in the validation group. The ROC curves were used to show the diagnostic ability of DNA methylation signature with 1-Specificity and sensitivity. The x-axis shows 1-specificity and y-axis shows sensitivity.

In order to explore the association between the risk score and identified DNA methylation signature, the clustering analysis map of methylation value in the DNA methylation signature sites was performed (Fig 1). In the risk score calculation, the patients were dichotomized into either low risk or the high risk group. In the training group, a highly significant difference was observed between the high risk and the low risk group (p = 1.1e-06), which was shown in Fig 2. When the same DNA methylation signature equation was applied to the validation group, a similar significant difference was also observed between the high risk and the low risk group (p = 0.054), which was shown in Fig 3. Additionally, we performed 5 years’ survival analysis of DNA methylation signature by ROC and calculated the AUC to assess the discriminatory ability of DNA methylation signature in the training and validation group, respectively (Figs 4 and 5). The AUC of the DNA methylation signature in the training group was 0.8441558, and the validation was 0.8170732. Our result suggested that the DNA methylation signature could be the prognosis model for predicting the survival situation of AML.

### Identification of risk related DNA methylation sites

In order to identify the DNA methylation sites between patients with poor-risk and favorable-risk, the differentially methylated sites and CpG islands were analyzed based on the threshold of |Δβ|>0.2 and FDR<0.05. The result showed that 70 differentially methylated sites (64 hypermethylation and 6 hypomethylation sites) and 6 differentially methylated CpG islands (3 hypermethylation and 3 hypomethylation CpG islands) were identified. The top 20 differentially methylated sites in the poor-risk and favorable-risk group were shown in Table 4.

**Table 4:** The top 20 differentially methylated sites in the poor-risk and favorable-risk group.

Site	Gene	Island	Δβ	FDR
cg27321949	*LRPAP1*	chr4:3516456–3516844	0.597435476	4.82581E-17
cg04857395	*LRPAP1*	chr4:3516456–3516844	0.603433175	4.82581E-17
cg25278298	*MAEA*	chr4:1303490–1303835	0.41009799	6.33572E-17
cg24973755	*MAEA*	chr4:1304768–1305114	0.539319729	6.33572E-17
cg06466348	*ADCY7*		0.529336818	4.87433E-16
cg09936008	*ZNF213*	chr16:3190765–3191389	0.522768451	3.94334E-15
cg01360627	*TNF*	chr6:31548436–31549277	0.556695472	5.62805E-15
cg18349022	*ZNF498*		0.574523405	6.55958E-15
cg13458384	*SARDH*	chr9:136567684–136568146	0.410375297	1.34354E-14
cg17741993	*TNF*	chr6:31548436–31549277	0.587861991	1.34354E-14
cg25197194	*CCDC48*		0.55303526	3.2255E-14
cg26996656			0.558266306	3.37347E-14
cg17427926	*LOC283999*	chr17:76228110–76228380	0.468902001	8.05456E-14
cg02340818	*KIAA1688*	chr8:145806258–145806713	0.480393004	9.56514E-14
cg06250720			0.483657658	1.96516E-13
cg11227278	*KLHL29*	chr2:23749086–23749291	0.509376312	
cg17115419			0.493501781	
cg27049094	*HK2*		0.567439864	
cg01824603	*ARHGEF10*		0.511145159	
cg25605731	*CALR*	chr19:13056458–13057125	0.588599806	

Δβ: mean (β case) − mean (β normal)

FDR: false discovery rate

## Discussion

AML is the most common form of adult leukemia and the survival rate is very low [19–21]. Therefore, it is urgent to understand the pathological mechanism and find potential survival related genes in the development of AML. In this study, we found a DNA methylation signature involving *MYF6*, *RPTOR*, *MMP10*, *SH3PXD2B*, *VHL* and *GABBR1*, which could be a valuable tool in guiding treatment decisions for AML.

Myogenic factor 6 (*MYF6*, also called MRF4 or Herculin) is expressed in skeletal muscle and is related to myogenesis [22–25]. It is found that the *MYF6* is a differentially methylation gene in plasma cf-DNA in the different stage of hepatocellular carcinoma development [26]. In addition, the methylation frequency of *MYF6* in stage I non-small cell lung cancer is obviously higher than that of non-cancerous lung disease control, which suggested that *MYF6* could offer a specificity and a sensitivity in the stage I non-small cell lung cancer diagnosis [27]. Thus it can be seen that *MYF6* methylation was associated with the development of cancer. In this study, we first found the DNA methylation of *MYF6* in the blood of AML. Our result showed that *MYF6* was significantly associated with survival time of AML and could be a diagnostic and prognostic marker of AML.

Regulatory associated protein of MTOR complex 1 (*RPTOR*) is a signal transduction gene important for hematopoiesis [28]. *RPTOR*, mechanistic target of rapamycin kinase and MTOR associated protein (mTOR), LST8 homolog (MLST8) constitute the core subunits of the mammalian TORC1 complex which play an important role in controlling cell growth, survival and metabolism and is often deregulated in cancer [29–32]. It is reported that *RPTOR* is hypermethylated in human hepatocytes [33]. Previous reports have demonstrated that *RPTOR* is methylated gene in breast tumors [34, 35]. This suggested that *RPTOR* methylation may play an important role in the process of cancer. In the present study, we found that *RPTOR* was methylated in the blood of AML and significantly associated with the survival time of AML patients. Our result suggested that *RPTOR* played a crucial role in AML and may be a diagnostic and prognostic marker in the process of AML.

Matrix metallopeptidase 10 (MMP10), an enzyme promoting angiogenesis, promotes cell growth and invasion, and exerts anti-apoptotic property in vitro [36, 37]. It is noted that MMP10 is essential to the tumor microenvironment. In colorectal cancer, MMP10 is up-regulated in cancerous tissue and adversely associated with patients survival [38, 39]. In cutaneous melanoma, *MMP10* is potentially modified by modification of histone acetylation [40]. In head and neck squamous cell cancer, *MMP10* is a differentially methylated gene in radiation-sensitive and -resistant tumors [41]. In immature teratomas, reduced methylation of *MMP10* significantly enriched in axonal guidance signaling pathway [42]. Previous reports indicated that the epigenomics changes of *MMP10*, especially DNA methylation may be involved in the occurrence of cancer. Herein, we found the methylation of *MMP10* in the blood of AML. Moreover, *MMP10* was related to the survivability of AML patients. Our result may provide a new field in the diagnosis and prognosis of AML.

SH3 and PX domains 2B (SH3PXD2B) is associated with growth and production. It is found that SH3PXD2B is involved in the protein binding, cytoplasm and cell junction in hepatocellular carcinoma [43]. In addition, *SH3PXD2B* positively regulates the differentiation of fat cell and shows differences in DNA methylation in gene body or intergenic region [44, 45]. However, there are no reports about the relationship between *SH3PXD2B* methylation and cancer. In this study, we first found methylated *SH3PXD2B* in the blood of AML. Moreover, it was observably associated with the survival time of AML patients. In a word, *SH3PXD2B* may play a crucial role in AML and could be a diagnostic and prognostic marker in the development of AML.

The gene product of the Von Hippel-Lindau tumor suppressor (*VHL*) plays a key role in regulation of metabolic genes expression, erythropoiesis, angiogenesis, proliferation and apoptosis. VHL-associated tumors are highly vascularized and overproduce VEGF [46]. It is indicated that *VHL* promoter hypermethylation may play an important role in pheochromocytoma and abdominal paraganglioma development [47]. Herman and Zhong et al found that *VHL* was a methylated gene in renal carcinoma cell lines [48, 49]. Hypomethylation of *VHL* has been reported in head and neck cancer and lung squamous carcinoma [50, 51]. Thus, *VHL* functions as a methylated gene in different cancers. It is pointed out that the mutations in the *VHL* gene are involved in the pathogenesis of AML [46]. Herein, we also found the relationship between *VHL* and AML. Our result showed that *VHL* was methylated and was significantly associated with survival time of AML patients.

Gamma-aminobutyric acid type B receptor subunit 1 (*GABBR1*) encodes the G protein-coupled receptor that can form the heterodimer with GABAB receptor 2, which triggering the proliferation, differentiation and migration of cancer cells. It is showed that multiple loci of *GABBR1* within 6p21.3 are related to nasopharyngeal carcinoma [52, 53]. *GABBR1* is a different methylated gene in plasma cf-DNA in different early stage of hepatocellular carcinoma development [26]. In addition, *GABBR1* is associated with the survival time of patients with gastric cancer [54]. It is also a survival-associated methylation marker for oral squamous cell carcinoma [55]. Thus it can be seen that *GABBR1* methylation play a crucial role in various cancers. In the present study, we found that *GABBR1* was methylated and associated with AML survival. It suggested that *GABBR1* may be a survival-associated methylation marker for AML.

Previous studies have reported that the 5-year survival rate was 55% for patients with favorable cytogenetics, 24% for patients with intermediate risk, and 5% for patients with poor-risk cytogenetics [56]. Thus it can be seen that the survival time was associated with AML risk. In this study, we identified numbers of survival-associated differentially methylated genes such as *LRPAP1*, *MAEA* and *TNF* between poor-risk and favorable-risk patients.

LDL receptor related protein associated protein 1 (*LRPAP1*) is a gene that involved in the cell proliferation in cancer [57]. It is noted that *LRPAP1* is a biologically relevant gene found in leukemia and was associated with different biological processes including cell apoptosis, signaling pathway and cell cycle checkpoint [58, 59]. Macrophage erythroblast attacher (MAEA) is a 36-kD transmembrane protein that expressed by erythroblasts and macrophage cells and plays a crucial role in hematopoiesis [60, 61]. It is reported that *MAEA* is a differentially methylated gene in Type-2 diabetes and idiopathic pulmonary fibrosis [62, 63]. Tumor necrosis factor (TNF) is a proinflammatory cytokine. It has been identified that TNF is elevated in serum of patients with aplastic anemia and myelodysplastic syndromes, suggesting that the hematopoietic repressive activity of TNF may contribute to the cytopenic phenotype of these patients [64–67]. Interestingly, TNF levels are significantly higher in the peripheral blood of AML patients of M3, M4, and M5 subtypes when compared with healthy donors [68]. Furthermore, the increased level of TNF is associated with poor prognosis of patients with AML, especially older adults [7, 69–71]. Our result further indicated the important role of *LRPAP1*, *MAEA* and *TNF* in AML.

## Conclusions

In a word, we have identified and successfully validated a DNA methylation signature in patients with AML. This signature adds to the potential predictive role in the survival time of AML patients. Utilization of a prognostic DNA methylation signature would enrich for potentially sensitive patients, thereby improving clinical outcome for future patients with AML. However, there is a limitation to our study. In the present study, we didn’t perform the deeper mechanism study based on the identified methylated genes. Some animal model and cell experiments are further needed to explore the potential mechanism of AML.

## References

[1] Ryningen A, Wergeland L, Glenjen N, Gjertsen BT, Bruserud O. In vitro crosstalk between fibroblasts and native human acute myelogenous leukemia (AML) blasts via local cytokine networks results in increased proliferation and decreased apoptosis of AML cells as well as increased levels of proangiogenic Interleukin 8. Leukemia research. 2005;29(2):185–96. Epub 2004/12/21. doi: 10.1016/j.leukres.2004.06.008 . 15607368 10.1016/j.leukres.2004.06.008

[2] Horwitz M, Goode EL, Jarvik GP. Anticipation in familial leukemia. American journal of human genetics. 1996;59(5):990–8. Epub 1996/11/01. . 8900225 PMC1914843

[3] Evans DI, Steward JK. Down’s syndrome and leukaemia. Lancet (London, England). 1972;2(7790):1322 Epub 1972/12/16. . 4117858 10.1016/s0140-6736(72)92704-3

[4] Yoshinaga S, Mabuchi K, Sigurdson AJ, Doody MM, Ron E. Cancer risks among radiologists and radiologic technologists: review of epidemiologic studies. Radiology. 2004;233(2):313–21. Epub 2004/09/18. doi: 10.1148/radiol.2332031119 . 15375227 10.1148/radiol.2332031119

[5] Austin H, Delzell E, Cole P. Benzene and leukemia. A review of the literature and a risk assessment. American journal of epidemiology. 1988;127(3):419–39. Epub 1988/03/01. . 3277397 10.1093/oxfordjournals.aje.a114820

[6] Dohner H, Weisdorf DJ, Bloomfield CD. Acute Myeloid Leukemia. The New England journal of medicine. 2015;373(12):1136–52. Epub 2015/09/17. doi: 10.1056/NEJMra1406184 . 26376137 10.1056/NEJMra1406184

[7] Ishikawa F, Yoshida S, Saito Y, Hijikata A, Kitamura H, Tanaka S, et al Chemotherapy-resistant human AML stem cells home to and engraft within the bone-marrow endosteal region. Nature biotechnology. 2007;25(11):1315–21. Epub 2007/10/24. doi: 10.1038/nbt1350 . 17952057 10.1038/nbt1350

[8] Shuichi M. [Guideline for AML]. [Rinshō ketsueki] The Japanese journal of clinical hematology. 2013;54(10):1633–42. 24064812

[9] Levine RL. Molecular pathogenesis of AML: translating insights to the clinic. Best practice & research Clinical haematology. 2013;26(3):245–8. Epub 2013/12/07. doi: 10.1016/j.beha.2013.10.003 . 24309525 10.1016/j.beha.2013.10.003 PMC3869618

[10] Al-Ali HK, Jaekel N, Niederwieser D. The role of hypomethylating agents in the treatment of elderly patients with AML. Journal of geriatric oncology. 2014;5(1):89–105. Epub 2014/02/04. doi: 10.1016/j.jgo.2013.08.004 . 24484723 10.1016/j.jgo.2013.08.004

[11] Esteller M. Epigenetics in cancer. The New England journal of medicine. 2008;358(11):1148–59. Epub 2008/03/14. doi: 10.1056/NEJMra072067 . 18337604 10.1056/NEJMra072067

[12] Plass C, Oakes C, Blum W, Marcucci G. Epigenetics in acute myeloid leukemia. Seminars in oncology. 2008;35(4):378–87. Epub 2008/08/12. doi: 10.1053/j.seminoncol.2008.04.008 . 18692688 10.1053/j.seminoncol.2008.04.008 PMC3463865

[13] Jiang Y, Dunbar A, Gondek LP, Mohan S, Rataul M, O’Keefe C, et al Aberrant DNA methylation is a dominant mechanism in MDS progression to AML. Blood. 2009;113(6):1315–25. Epub 2008/10/04. doi: 10.1182/blood-2008-06-163246 . 18832655 10.1182/blood-2008-06-163246 PMC2637194

[14] Figueroa ME, Lugthart S, Li Y, Erpelinck-Verschueren C, Deng X, Christos PJ, et al DNA methylation signatures identify biologically distinct subtypes in acute myeloid leukemia. Cancer cell. 2010;17(1):13–27. Epub 2010/01/12. doi: 10.1016/j.ccr.2009.11.020 . 20060365 10.1016/j.ccr.2009.11.020 PMC3008568

[15] Bullinger L, Ehrich M, Dohner K, Schlenk RF, Dohner H, Nelson MR, et al Quantitative DNA methylation predicts survival in adult acute myeloid leukemia. Blood. 2010;115(3):636–42. Epub 2009/11/12. doi: 10.1182/blood-2009-03-211003 . 19903898 10.1182/blood-2009-03-211003

[16] Deneberg S, Grovdal M, Karimi M, Jansson M, Nahi H, Corbacioglu A, et al Gene-specific and global methylation patterns predict outcome in patients with acute myeloid leukemia. Leukemia. 2010;24(5):932–41. Epub 2010/03/20. doi: 10.1038/leu.2010.41 . 20237504 10.1038/leu.2010.41

[17] Jost E, Schmid J, Wilop S, Schubert C, Suzuki H, Herman JG, et al Epigenetic inactivation of secreted Frizzled-related proteins in acute myeloid leukaemia. British journal of haematology. 2008;142(5):745–53. Epub 2008/06/10. doi: 10.1111/j.1365-2141.2008.07242.x . 18537968 10.1111/j.1365-2141.2008.07242.x

[18] Tao YF, Fang F, Hu SY, Lu J, Cao L, Zhao WL, et al Hypermethylation of the GATA binding protein 4 (GATA4) promoter in Chinese pediatric acute myeloid leukemia. BMC cancer. 2015;15:756 Epub 2015/10/23. doi: 10.1186/s12885-015-1760-5 . 26490736 10.1186/s12885-015-1760-5 PMC4618362

[19] Larkin K, Blum W. Novel therapies in AML: reason for hope or just hype? American Society of Clinical Oncology educational book American Society of Clinical Oncology Meeting. 2014:e341–51. Epub 2014/05/27. doi: 10.14694/EdBook_AM.2014.34.e341 . 24857123 10.14694/EdBook_AM.2014.34.e341

[20] Kohgo Y, Inamura J, Shindo M. [Molecular target drugs for AML—current state and prospects for the future]. Nihon rinsho Japanese journal of clinical medicine. 2014;72(6):1063–7. Epub 2014/07/16. . 25016805

[21] Zeijlemaker W, Gratama JW, Schuurhuis GJ. Tumor heterogeneity makes AML a "moving target" for detection of residual disease. Cytometry Part B, Clinical cytometry. 2014;86(1):3–14. Epub 2013/10/24. 10.1002/cyto.b.21134 24151248

[22] Lassar AB, Paterson BM, Weintraub H. Transfection of a DNA locus that mediates the conversion of 10T1/2 fibroblasts to myoblasts. Cell. 1986;47(5):649–56. Epub 1986/12/05. . 2430720 10.1016/0092-8674(86)90507-6

[23] Wright WE, Sassoon DA, Lin VK. Myogenin, a factor regulating myogenesis, has a domain homologous to MyoD. Cell. 1989;56(4):607–17. Epub 1989/02/24. . 2537150 10.1016/0092-8674(89)90583-7

[24] Braun T, Winter B, Bober E, Arnold HH. Transcriptional activation domain of the muscle-specific gene-regulatory protein myf5. Nature. 1990;346(6285):663–5. Epub 1990/08/16. doi: 10.1038/346663a0 . 2385294 10.1038/346663a0

[25] Braun T, Bober E, Winter B, Rosenthal N, Arnold HH. Myf-6, a new member of the human gene family of myogenic determination factors: evidence for a gene cluster on chromosome 12. The EMBO journal. 1990;9(3):821–31. Epub 1990/03/01. . 2311584 10.1002/j.1460-2075.1990.tb08179.x PMC551742

[26] Zhao Y, Xue F, Sun J, Guo S, Zhang H, Qiu B, et al Genome-wide methylation profiling of the different stages of hepatitis B virus-related hepatocellular carcinoma development in plasma cell-free DNA reveals potential biomarkers for early detection and high-risk monitoring of hepatocellular carcinoma. Clinical epigenetics. 2014;6(1):30 Epub 2014/01/01. doi: 10.1186/1868-7083-6-30 . 25859288 10.1186/1868-7083-6-30 PMC4391300

[27] Zhao Y, Zhou H, Ma K, Sun J, Feng X, Geng J, et al Abnormal methylation of seven genes and their associations with clinical characteristics in early stage non-small cell lung cancer. Oncology letters. 2013;5(4):1211–8. Epub 2013/04/20. doi: 10.3892/ol.2013.1161 . 23599765 10.3892/ol.2013.1161 PMC3629069

[28] Kaushansky A, Kaushansky K. Systems biology of megakaryocytes. Advances in experimental medicine and biology. 2014;844:59–84. Epub 2014/12/07. doi: 10.1007/978-1-4939-2095-2_4 . 25480637 10.1007/978-1-4939-2095-2_4

[29] Hay N, Sonenberg N. Upstream and downstream of mTOR. Genes & development. 2004;18(16):1926–45. Epub 2004/08/18. doi: 10.1101/gad.1212704 . 15314020 10.1101/gad.1212704

[30] Sabatini DM. mTOR and cancer: insights into a complex relationship. Nature reviews Cancer. 2006;6(9):729–34. Epub 2006/08/18. doi: 10.1038/nrc1974 . 16915295 10.1038/nrc1974

[31] Sengupta S, Peterson TR, Sabatini DM. Regulation of the mTOR complex 1 pathway by nutrients, growth factors, and stress. Molecular cell. 2010;40(2):310–22. Epub 2010/10/23. doi: 10.1016/j.molcel.2010.09.026 . 20965424 10.1016/j.molcel.2010.09.026 PMC2993060

[32] Zoncu R, Efeyan A, Sabatini DM. mTOR: from growth signal integration to cancer, diabetes and ageing. Nature reviews Molecular cell biology. 2011;12(1):21–35. Epub 2010/12/16. doi: 10.1038/nrm3025 . 21157483 10.1038/nrm3025 PMC3390257

[33] Wolters J, van Breda S, Claessen S, de Kok T, Kleinjans J. Data on novel DNA methylation changes induced by valproic acid in human hepatocytes. Data in brief. 2018;16:161–71. Epub 2017/12/05. doi: 10.1016/j.dib.2017.11.031 . 29201983 10.1016/j.dib.2017.11.031 PMC5702865

[34] Comprehensive molecular portraits of human breast tumours. Nature. 2012;490(7418):61–70. Epub 2012/09/25. doi: 10.1038/nature11412 . 23000897 10.1038/nature11412 PMC3465532

[35] Fleischer T, Frigessi A, Johnson KC, Edvardsen H, Touleimat N, Klajic J, et al Genome-wide DNA methylation profiles in progression to in situ and invasive carcinoma of the breast with impact on gene transcription and prognosis. Genome biology. 2014;15(8):435 Epub 2014/08/26. doi: 10.1186/PREACCEPT-2333349012841587 . 25146004 10.1186/s13059-014-0435-x PMC4165906

[36] Huang L, Xu Y, Cai G, Guan Z, Cai S. Downregulation of S100A4 expression by RNA interference suppresses cell growth and invasion in human colorectal cancer cells. Oncology reports. 2012;27(4):917–22. Epub 2011/12/28. doi: 10.3892/or.2011.1598 . 22200787 10.3892/or.2011.1598 PMC3583610

[37] Meyer E, Vollmer JY, Bovey R, Stamenkovic I. Matrix metalloproteinases 9 and 10 inhibit protein kinase C-potentiated, p53-mediated apoptosis. Cancer research. 2005;65(10):4261–72. Epub 2005/05/19. doi: 10.1158/0008-5472.CAN-04-2908 . 15899818 10.1158/0008-5472.CAN-04-2908

[38] Masaki T, Matsuoka H, Sugiyama M, Abe N, Goto A, Sakamoto A, et al Matrilysin (MMP-7) as a significant determinant of malignant potential of early invasive colorectal carcinomas. British journal of cancer. 2001;84(10):1317–21. Epub 2001/05/18. doi: 10.1054/bjoc.2001.1790 . 11355941 10.1054/bjoc.2001.1790 PMC2363635

[39] Ting WC, Chen LM, Pao JB, Yang YP, You BJ, Chang TY, et al Genetic polymorphisms of matrix metalloproteinases and clinical outcomes in colorectal cancer patients. International journal of medical sciences. 2013;10(8):1022–7. Epub 2013/06/27. doi: 10.7150/ijms.6686 . 23801889 10.7150/ijms.6686 PMC3691801

[40] Kim SH, Ahn S, Han JW, Lee HW, Lee HY, Lee YW, et al Apicidin is a histone deacetylase inhibitor with anti-invasive and anti-angiogenic potentials. Biochemical and biophysical research communications. 2004;315(4):964–70. Epub 2004/02/27. doi: 10.1016/j.bbrc.2004.01.149 14985106 10.1016/j.bbrc.2004.01.149

[41] Chen X, Liu L, Mims J, Punska EC, Williams KE, Zhao W, et al Analysis of DNA methylation and gene expression in radiation-resistant head and neck tumors. Epigenetics. 2015;10(6):545–61. Epub 2015/05/12. doi: 10.1080/15592294.2015.1048953 . 25961636 10.1080/15592294.2015.1048953 PMC4622425

[42] Amatruda JF, Ross JA, Christensen B, Fustino NJ, Chen KS, Hooten AJ, et al DNA methylation analysis reveals distinct methylation signatures in pediatric germ cell tumors. BMC cancer. 2013;13:313 Epub 2013/06/29. doi: 10.1186/1471-2407-13-313 . 23806198 10.1186/1471-2407-13-313 PMC3701494

[43] Wang C, Ren R, Hu H, Tan C, Han M, Wang X, et al MiR-182 is up-regulated and targeting Cebpa in hepatocellular carcinoma. Chinese journal of cancer research = Chung-kuo yen cheng yen chiu. 2014;26(1):17–29. Epub 2014/03/22. doi: 10.3978/j.issn.1000-9604.2014.01.01 . 24653623 10.3978/j.issn.1000-9604.2014.01.01 PMC3937760

[44] Fan C, Dong H, Yan K, Shen W, Wang C, Xia L, et al Genome-wide screen of promoter methylation identifies novel markers in diet-induced obese mice. Nutricion hospitalaria. 2014;30(1):42–52. Epub 2014/08/20. doi: 10.3305/nh.2014.30.1.7521 . 25137261 10.3305/nh.2014.30.1.7521

[45] Wang Y, Ma C, Sun Y, Li Y, Kang L, Jiang Y. Dynamic transcriptome and DNA methylome analyses on longissimus dorsi to identify genes underlying intramuscular fat content in pigs. BMC genomics. 2017;18(1):780 Epub 2017/10/14. doi: 10.1186/s12864-017-4201-9 . 29025412 10.1186/s12864-017-4201-9 PMC5639760

[46] Labno-Kirszniok K, Nieszporek T, Wiecek A, Helbig G, Lubinski J. Acute myeloid leukemia in a 38-year-old hemodialyzed patient with von Hippel-Lindau disease. Hereditary cancer in clinical practice. 2013;11(1):11 Epub 2013/08/24. doi: 10.1186/1897-4287-11-11 . 23968328 10.1186/1897-4287-11-11 PMC3846582

[47] Andreasson A, Kiss NB, Caramuta S, Sulaiman L, Svahn F, Backdahl M, et al The VHL gene is epigenetically inactivated in pheochromocytomas and abdominal paragangliomas. Epigenetics. 2013;8(12):1347–54. Epub 2013/10/24. doi: 10.4161/epi.26686 . 24149047 10.4161/epi.26686 PMC3933494

[48] Herman JG, Latif F, Weng Y, Lerman MI, Zbar B, Liu S, et al Silencing of the VHL tumor-suppressor gene by DNA methylation in renal carcinoma. Proceedings of the National Academy of Sciences of the United States of America. 1994;91(21):9700–4. Epub 1994/10/11. . 7937876 10.1073/pnas.91.21.9700 PMC44884

[49] Zhong CX, Mass MJ. Both hypomethylation and hypermethylation of DNA associated with arsenite exposure in cultures of human cells identified by methylation-sensitive arbitrarily-primed PCR. Toxicology letters. 2001;122(3):223–34. Epub 2001/08/08. . 11489357 10.1016/s0378-4274(01)00365-4

[50] Comprehensive genomic characterization of squamous cell lung cancers. Nature. 2012;489(7417):519–25. Epub 2012/09/11. doi: 10.1038/nature11404 . 22960745 10.1038/nature11404 PMC3466113

[51] Poage GM, Butler RA, Houseman EA, Mcclean MD, Nelson HH, Christensen BC, et al Identification of an epigenetic profile classifier that is associated with survival in head and neck cancer. Cancer research. 2012;72(11):2728–37. doi: 10.1158/0008-5472.CAN-11-4121-T 22507853 10.1158/0008-5472.CAN-11-4121-T PMC3650639

[52] KaPo Su, WenHui Chang, KaiPing Tsang, et al Genome-wide Association Study Reveals Multiple Nasopharyngeal Carcinoma-Associated Loci within the HLA Region at Chromosome 6p21.3. American journal of human genetics. 2009;85(2):194–203. doi: 10.1016/j.ajhg.2009.07.007 19664746 10.1016/j.ajhg.2009.07.007 PMC2725267

[53] Tang M, Lautenberger JA, Gao X, Sezgin E, Hendrickson SL, Troyer JL, et al The principal genetic determinants for nasopharyngeal carcinoma in China involve the HLA class I antigen recognition groove. PLoS genetics. 2012;8(11):e1003103 Epub 2012/12/05. doi: 10.1371/journal.pgen.1003103 . 23209447 10.1371/journal.pgen.1003103 PMC3510037

[54] Wang P, Wang Y, Hang B, Zou X, Mao JH. A novel gene expression-based prognostic scoring system to predict survival in gastric cancer. Oncotarget. 2016;7(34):55343–51. Epub 2016/07/16. doi: 10.18632/oncotarget.10533 . 27419373 10.18632/oncotarget.10533 PMC5342421

[55] Langevin SM, Butler RA, Eliot M, Pawlita M, Maccani JZ, McClean MD, et al Novel DNA methylation targets in oral rinse samples predict survival of patients with oral squamous cell carcinoma. Oral oncology. 2014;50(11):1072–80. Epub 2014/09/23. doi: 10.1016/j.oraloncology.2014.08.015 . 25242135 10.1016/j.oraloncology.2014.08.015 PMC4254027

[56] Byrd JC, Mrozek K, Dodge RK, Carroll AJ, Edwards CG, Arthur DC, et al Pretreatment cytogenetic abnormalities are predictive of induction success, cumulative incidence of relapse, and overall survival in adult patients with de novo acute myeloid leukemia: results from Cancer and Leukemia Group B (CALGB 8461). Blood. 2002;100(13):4325–36. Epub 2002/10/24. doi: 10.1182/blood-2002-03-0772 . 12393746 10.1182/blood-2002-03-0772

[57] Sinha AU, Kaimal V, Chen J, Jegga AG. Dissecting microregulation of a master regulatory network. BMC genomics. 2008;9:88 Epub 2008/02/26. doi: 10.1186/1471-2164-9-88 . 18294391 10.1186/1471-2164-9-88 PMC2289817

[58] Chen AH, Tsau YW, Lin CH. Novel methods to identify biologically relevant genes for leukemia and prostate cancer from gene expression profiles. BMC genomics. 2010;11:274 Epub 2010/05/04. doi: 10.1186/1471-2164-11-274 . 20433712 10.1186/1471-2164-11-274 PMC2873479

[59] Kushwaha G, Dozmorov M, Wren JD, Qiu J, Shi H, Xu D. Hypomethylation coordinates antagonistically with hypermethylation in cancer development: a case study of leukemia. Human genomics. 2016;10 Suppl 2:18 Epub 2016/07/28. doi: 10.1186/s40246-016-0071-5 . 27461342 10.1186/s40246-016-0071-5 PMC4965721

[60] Hanspal M, Hanspal JS. The association of erythroblasts with macrophages promotes erythroid proliferation and maturation: a 30-kD heparin-binding protein is involved in this contact. Blood. 1994;84(10):3494–504. Epub 1994/11/15. . 7949103

[61] Hanspal M, Smockova Y, Uong Q. Molecular identification and functional characterization of a novel protein that mediates the attachment of erythroblasts to macrophages. Blood. 1998;92(8):2940–50. Epub 1998/10/09. . 9763581

[62] Benton MC, Johnstone A, Eccles D, Harmon B, Hayes MT, Lea RA, et al An analysis of DNA methylation in human adipose tissue reveals differential modification of obesity genes before and after gastric bypass and weight loss. Genome biology. 2015;16:8 Epub 2015/02/05. doi: 10.1186/s13059-014-0569-x . 25651499 10.1186/s13059-014-0569-x PMC4301800

[63] Rabinovich EI, Kapetanaki MG, Steinfeld I, Gibson KF, Pandit KV, Yu G, et al Global methylation patterns in idiopathic pulmonary fibrosis. PloS one. 2012;7(4):e33770 Epub 2012/04/17. doi: 10.1371/journal.pone.0033770 . 22506007 10.1371/journal.pone.0033770 PMC3323629

[64] Molnar L, Berki T, Hussain A, Nemeth P, Losonczy H. Detection of TNFalpha expression in the bone marrow and determination of TNFalpha production of peripheral blood mononuclear cells in myelodysplastic syndrome. Pathology oncology research: POR. 2000;6(1):18–23. Epub 2000/04/05. . 10749583 10.1007/BF03032653

[65] Dybedal I, Bryder D, Fossum A, Rusten LS, Jacobsen SE. Tumor necrosis factor (TNF)-mediated activation of the p55 TNF receptor negatively regulates maintenance of cycling reconstituting human hematopoietic stem cells. Blood. 2001;98(6):1782–91. Epub 2001/09/06. . 11535512 10.1182/blood.v98.6.1782

[66] Dufour C, Corcione A, Svahn J, Haupt R, Poggi V, Beka’ssy AN, et al TNF-alpha and IFN-gamma are overexpressed in the bone marrow of Fanconi anemia patients and TNF-alpha suppresses erythropoiesis in vitro. Blood. 2003;102(6):2053–9. Epub 2003/05/17. doi: 10.1182/blood-2003-01-0114 . 12750172 10.1182/blood-2003-01-0114

[67] Lv L, Kerzic P, Lin G, Schnatter AR, Bao L, Yang Y, et al The TNF-alpha 238A polymorphism is associated with susceptibility to persistent bone marrow dysplasia following chronic exposure to benzene. Leukemia research. 2007;31(11):1479–85. Epub 2007/03/21. doi: 10.1016/j.leukres.2007.01.014 . 17367855 10.1016/j.leukres.2007.01.014

[68] Volk A, Li J, Xin J, You D, Zhang J, Liu X, et al Co-inhibition of NF-kappaB and JNK is synergistic in TNF-expressing human AML. The Journal of experimental medicine. 2014;211(6):1093–108. Epub 2014/05/21. doi: 10.1084/jem.20130990 . 24842373 10.1084/jem.20130990 PMC4042653

[69] Rao AV, Valk PJ, Metzeler KH, Acharya CR, Tuchman SA, Stevenson MM, et al Age-specific differences in oncogenic pathway dysregulation and anthracycline sensitivity in patients with acute myeloid leukemia. Journal of clinical oncology: official journal of the American Society of Clinical Oncology. 2009;27(33):5580–6. Epub 2009/10/28. doi: 10.1200/jco.2009.22.2547 . 19858393 10.1200/JCO.2009.22.2547

[70] Hoang T, Haman A, Goncalves O, Letendre F, Mathieu M, Wong GG, et al Interleukin 1 enhances growth factor-dependent proliferation of the clonogenic cells in acute myeloblastic leukemia and of normal human primitive hemopoietic precursors. The Journal of experimental medicine. 1988;168(2):463–74. Epub 1988/08/01. . 3261773 10.1084/jem.168.2.463 PMC2189011

[71] Cozzolino F, Rubartelli A, Aldinucci D, Sitia R, Torcia M, Shaw A, et al Interleukin 1 as an autocrine growth factor for acute myeloid leukemia cells. Proceedings of the National Academy of Sciences of the United States of America. 1989;86(7):2369–73. Epub 1989/04/01. . 2522658 10.1073/pnas.86.7.2369 PMC286914

